# Reinforcement Learning-Based Golf Swing Correction Framework Incorporating Temporal Rhythm and Kinematic Stability

**DOI:** 10.3390/s26020392

**Published:** 2026-01-07

**Authors:** Dong-Jun Lee, Young-Been Noh, Jeongeun Byun, Kwang-Il Hwang

**Affiliations:** 1 Department of Embedded Systems Engineering, College of Information Technology, Incheon National University, Incheon 22012, Republic of Korea; snow340@naver.com (D.-J.L.); noyoungbin40@gmail.com (Y.-B.N.); 2 Research Center for Technology Commercialization, Korea Institute of Science & Technology Information (KISTI), Seoul 02456, Republic of Korea; jebyun@kisti.re.kr

**Keywords:** golf swing correction, reinforcement learning, Proximal Policy Optimization, temporal rhythm, Velocity-DTW, pose-based motion analysis

## Abstract

Accurate golf swing correction requires modeling not only static pose deviations but also temporal rhythm and biomechanical stability throughout the swing sequence. Most existing pose-based approaches rely on frame-wise similarity and therefore fail to capture timing, velocity transitions, and coordinated joint dynamics. This study proposes a reinforcement learning-based framework that generates frame-level corrective motions by formulating swing correction as a sequential decision-making problem optimized via Proximal Policy Optimization (PPO). A multi-term reward function is designed to integrate geometric pose accuracy, incremental correction improvement, hip-centered stability, and temporal rhythm consistency measured using a Velocity-DTW metric. Experiments conducted with swing sequences from one professional and five amateur golfers demonstrate that the proposed method produces smoother and more temporally coherent corrections than static pose–based baselines. In particular, rhythm-aware rewards substantially improve the motion of highly dynamic joints, such as the wrists and shoulders, while preserving lower-body stability. Visual analyses further confirm that the corrected trajectories follow expert patterns in both spatial alignment and timing. These results indicate that explicitly incorporating temporal rhythm within a reinforcement learning framework is essential for realistic and effective swing correction. The proposed method provides a principled foundation for automated, expert-level coaching systems in golf and other dynamic sports requiring temporally coordinated whole-body motion.

## 1. Introduction

Human motion analysis has been extensively studied for applications in sports training, rehabilitation, and health monitoring. Early approaches relied on depth-based sensing or handcrafted representations to analyze body movement patterns [1]. With the advancement of deep learning, pose embedding methods [2] and convolutional backbone–based 2D pose estimation frameworks [3] have enabled more robust extraction of human joint information from monocular images. Recent pose estimation systems such as OpenPose [4], BlazePose [5], and bottom-up keypoint regression methods [6] have further improved accuracy and real-time performance, facilitating large-scale motion analysis in unconstrained environments.

Beyond pose extraction, motion representation and alignment techniques have been actively explored to enable reliable comparison of pose sequences across subjects and execution styles. Normalized pose representations reduce variations caused by body size, camera viewpoint, and execution speed, thereby improving alignment robustness [7]. Building on such representations, deep metric learning-based motion similarity evaluation [8] and lightweight contrastive pose sequence alignment approaches [9] have demonstrated improved performance in comparing human motion sequences. However, these methods predominantly focus on evaluating similarity rather than actively correcting motion in a temporally coherent manner.

In sports motion analysis, particularly in golf swing assessment, pose-based approaches have gained increasing attention due to their low-cost sensing requirements. Prior studies have proposed golf-specific pose refinement and embedding frameworks [10], deep learning-based swing similarity measurement methods [11], and posture-to-swing transformation models [12]. While effective in capturing geometric posture differences, most existing approaches rely on static or frame-wise similarity measures. Consequently, they struggle to model temporal rhythm, velocity transitions, and coordinated joint dynamics—key characteristics that distinguish expert-level golf swings from amateur executions.

Modeling and correcting human motion using reinforcement learning (RL) introduces additional challenges. Human movement patterns are inherently non-stationary, as individuals continuously adapt their behavior through practice, fatigue, and feedback. Moreover, sports motions such as golf swings exhibit substantial inter-subject variability and must satisfy biomechanical constraints to remain physically plausible. These characteristics make the direct application of RL to human behavior particularly challenging, as naive formulations may result in unstable learning dynamics or biomechanically implausible corrections.

Despite these challenges, sports motion correction remains an inherently sequential and decision-driven problem. Effective correction requires not only minimizing instantaneous pose discrepancies but also generating temporally consistent, phase-aware adjustments that evolve over the entire motion sequence. Such requirements are difficult to address using static or frame-independent formulations and naturally align with the reinforcement learning paradigm, in which motion correction can be formulated as a sequential decision-making process with long-term objectives.

Reinforcement learning provides a principled framework for modeling sequential decision-making problems and has been successfully applied to locomotion control, posture optimization, and biomechanical modeling [13,14]. Policy-gradient and actor–critic methods [15], particularly Proximal Policy Optimization (PPO) [16], enable stable learning in continuous action spaces and are well suited for incremental motion refinement. Nevertheless, the application of RL to pose-based sports motion correction remains relatively underexplored, especially in scenarios where temporal rhythm and biomechanical stability must be considered jointly.

To address temporal misalignment caused by variations in swing duration and execution speed, Dynamic Time Warping (DTW) has been widely adopted in sports motion analysis [17,18,19] and fitness exercise evaluation systems [20]. While DTW is effective for post hoc sequence alignment and similarity evaluation, it is typically applied as an offline matching tool and does not directly influence the motion correction process. As a result, DTW-based approaches lack the capability to generate corrective actions that simultaneously preserve spatial accuracy and temporal motion flow during execution.

Motivated by these limitations, this paper proposes a reinforcement learning-based golf swing correction framework that explicitly incorporates temporal rhythm and kinematic stability into the correction objective. Swing correction is formulated as a sequential decision-making problem in which an RL agent generates incremental, frame-level joint correction vectors that progressively guide the user’s motion toward expert trajectories. By emphasizing gradual correction rather than abrupt pose replacement, the proposed formulation mitigates non-stationarity and preserves motion continuity.

A key contribution of this work is the integration of velocity-based Dynamic Time Warping (Velocity-DTW) into the reinforcement learning reward function, enabling the agent to internalize expert swing rhythm directly during training rather than relying on post hoc alignment. In addition, hip-centered stability constraints are introduced to maintain biomechanically plausible center-of-mass behavior. Together, these reward components allow the agent to balance geometric pose accuracy, temporal rhythm consistency, and biomechanical stability within a unified learning framework.

Comprehensive experiments and analyses demonstrate that the proposed framework produces smoother and more temporally coherent corrections than static pose-based baselines, particularly for highly dynamic upper-body joints such as the shoulders and wrists. The results highlight the importance of explicitly modeling temporal structure in sports motion correction and establish reinforcement learning as an effective paradigm for expert-level golf swing coaching.

The remainder of this paper is organized as follows. Section 2 reviews prior work on human pose estimation, golf swing analysis, temporal alignment, and reinforcement learning for human motion modeling. Section 3 describes the proposed reinforcement learning-based swing correction framework, including data preprocessing, Markov decision process formulation, reward design, and policy optimization. Section 4 presents quantitative experimental results, including ablation studies and reward sensitivity analyses, followed by a detailed comparison of static and rhythm-aware correction behaviors. Section 5 provides visual analyses of major joints and discusses system-generated, actionable feedback derived from corrected trajectories. Finally, Section 6 summarizes the findings, discusses limitations, and outlines directions for future research.

## 2. Related Work

### 2.1. Human Pose Estimation and Motion Representation

Human pose estimation has evolved from depth-based skeleton-free approaches [1] to deep learning-based 2D pose estimation frameworks [2,3]. Recent methods emphasize robustness, efficiency, and real-time inference, exemplified by OpenPose [4], BlazePose [5], and bottom-up keypoint regression approaches [6]. These advances enable reliable pose extraction in unconstrained environments, forming the foundation for large-scale motion analysis.

Beyond pose extraction, motion representation and alignment techniques have been actively explored to enable reliable comparison of pose sequences across subjects and execution styles. In particular, normalized human pose features have been shown to effectively reduce variations caused by body size, camera viewpoint, and execution speed, thereby improving alignment robustness in human motion analysis [7]. Building on such representations, deep metric learning-based similarity models [8] and lightweight contrastive alignment approaches [9] further enhance pose sequence matching performance in diverse motion scenarios.

While these approaches are effective for motion comparison and evaluation, they are primarily designed for similarity assessment rather than for generating temporally coherent motion corrections over time.

### 2.2. Golf Swing Analysis and Pose-Based Similarity Measurement

Pose-based golf swing analysis has become increasingly popular due to the accessibility of monocular cameras. Existing studies propose pose refinement and embedding-based self-training systems [10], deep learning-based swing similarity measurement methods [11], and golf-specific posture modeling frameworks [12]. Biomechanical studies further emphasize the importance of coordinated upper–lower body motion, such as pelvis–thorax coupling, in effective golf swing execution [21].

Despite these advances, most golf swing analysis methods rely on static pose similarity or embedding distances computed on a frame-wise or phase-aligned basis. As a result, temporal rhythm, velocity transitions, and phase-to-phase motion dynamics are insufficiently modeled. Consequently, these approaches are limited in their ability to generate temporally coherent swing corrections, particularly for highly dynamic joints. Our previous work [22] introduced an automatic swing phase segmentation framework, which is adopted in this study for consistent phase-wise analysis.

### 2.3. Temporal Alignment and Dynamic Time Warping

Dynamic Time Warping (DTW) has been widely used for temporal alignment of motion capture and time-series data [17,18,19]. DTW-based methods have also been applied to fitness exercise evaluation systems [20] and multivariate time-series similarity matching. While effective for aligning sequences of different lengths, DTW is typically applied as an offline alignment or evaluation tool and does not directly influence the motion generation or correction process.

Recent studies highlight the importance of velocity-aware modeling for motion forecasting and temporal consistency [23], suggesting that temporal dynamics should be explicitly incorporated into learning objectives rather than treated as post-processing constraints. However, most DTW-based approaches still operate outside the correction loop and therefore lack the ability to produce adaptive, sequential corrections during motion execution.

### 2.4. Reinforcement Learning for Human Motion and Posture Control

Reinforcement learning has been successfully applied to human locomotion modeling [13], posture detection [14], and biomechanical optimization [24]. Policy-gradient and actor–critic methods [15,16,25,26] enable stable learning for continuous control tasks, while recent studies explore active pose estimation [27] and adaptive reward design [28].

In parallel, supervised and imitation learning approaches have been explored for motion prediction and reproduction. However, such methods typically rely on frame-aligned supervision or direct trajectory regression, which limits their flexibility in generating incremental, user-specific corrections under temporal variability.

Although reinforcement learning has demonstrated strong potential in modeling complex human motions, its application to sports-specific motion correction with explicit temporal rhythm and biomechanical stability constraints remains limited. This gap motivates the proposed framework, which integrates reinforcement learning with rhythm-aware and stability-aware reward design for golf swing correction.

## 3. Reinforcement Learning-Based Golf Swing Correction Framework

### 3.1. System Overview

**Figure 1:**
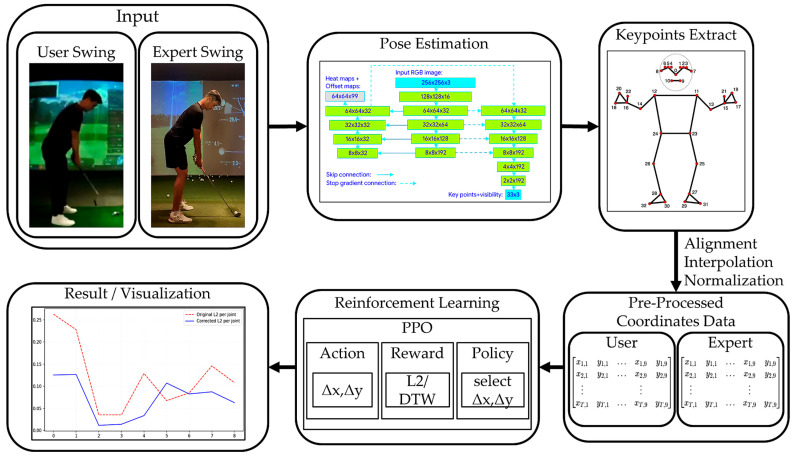
Overview of the proposed reinforcement learning-based golf swing correction framework. User and expert swing videos are processed through pose estimation and keypoint extraction, followed by temporal alignment and spatial normalization. A PPO-based reinforcement learning agent then generates joint-wise correction vectors to iteratively refine the user’s swing toward the expert motion, and the corrected results are visualized for qualitative and quantitative analysis.

Figure 1 illustrates the overall architecture of the proposed reinforcement learning-based golf swing correction framework. The system is designed as a sequential pipeline that transforms raw swing videos into corrected motion trajectories by integrating pose estimation, preprocessing, and reinforcement learning-based decision making.

The input to the framework consists of user swing and expert swing video sequences captured by a monocular camera. From each video, human joint keypoints are extracted using a pose estimation module. This module produces 2D joint coordinates for all relevant body parts, forming the basis for subsequent motion analysis and correction.

The extracted keypoints are then processed through a preprocessing stage, which includes temporal alignment, interpolation, and spatial normalization. Specifically, swing sequences are aligned on a phase-by-phase basis to account for differences in swing duration, while spatial normalization reduces inter-subject variability caused by body size and camera distance. As a result, both user and expert swings are converted into normalized, temporally aligned coordinate sequences, enabling reliable frame-level comparison.

Based on the preprocessed data, the swing correction task is formulated as a reinforcement learning (RL) problem. At each time step, the RL agent observes the current normalized pose state of the user and selects an action in the form of a joint-wise correction vector $\left(\Delta x,\Delta y\right)$. This correction vector incrementally modifies the user’s pose toward the expert reference rather than enforcing abrupt changes.

The policy is trained using Proximal Policy Optimization (PPO), which ensures stable learning in continuous action spaces. During training, the agent receives a reward composed of multiple terms, including pose accuracy, correction improvement rate, hip alignment stability, and temporal rhythm consistency based on Velocity-DTW. This reward formulation enables the agent to learn correction strategies that balance geometric accuracy with temporal coherence.

Finally, the corrected swing sequence is reconstructed from the accumulated correction vectors and provided as output. The system supports visualization and quantitative analysis, allowing users to compare original, corrected, and expert swings in terms of joint trajectories, error curves, and temporal alignment. Through this end-to-end pipeline, the proposed framework generates smooth, rhythm-aware swing corrections that progressively guide user motions toward expert-level patterns.

### 3.2. Data Preprocessing

To ensure a stable reinforcement learning environment, the motion data must satisfy several quality requirements, including temporal consistency across frames, reduced keypoint noise, and scale-invariant representation. The following preprocessing steps are designed to explicitly meet these requirements.

Data preprocessing is a crucial step for constructing a stable reinforcement learning environment, as golf swing data inherently exhibits variations in swing duration, execution speed, body size, and camera setup. To ensure consistent comparison and learning, the proposed framework performs phase-wise temporal alignment followed by hip-centered spatial normalization.

#### 3.2.1. Phase-Wise Temporal Alignment

A golf swing is a structured motion composed of distinct biomechanical phases. In this study, each swing sequence is segmented into eight canonical phases: address, takeaway, half, top, down-half, impact, follow-through, and finish. Let

(1) $$S^{u}=\left\{p_{1}^{u},p_{2}^{u},\ldots ,p_{T_{u}}^{u}\right\},\;S^{e}=\{p_{1}^{e},p_{2}^{e},\ldots ,p_{T_{e}}^{e}\}$$

denote the user and expert swing sequences, respectively, where $p_{t}\in R^{2N}$ represents the 2D coordinates of N joints at frame t.

Because the duration of each phase differs between users and experts, direct frame-wise comparison leads to temporal misalignment. To address this issue, each phase k is temporally aligned independently. Let $T_{k}^{u}$ and $T_{k}^{e}$ denote the number of frames in phase k for the user and expert swings, respectively. Linear interpolation is applied to resample the user sequence so that

(2) $$T_{k}^{u}\to T_{k}^{e}.$$

Specifically, the interpolated pose at the normalized time index τ∈[0,1] is computed as

(3) $$p_{k}^{u}(\tau )=(1-\alpha )\;p_{k}^{u}(t)+\alpha \;p_{k}^{u}(t+1),$$

where

(4) $$\alpha =\tau \cdot (T_{k}^{u}-1)-t,t=\lfloor \tau \cdot (T_{k}^{u}-1)\rfloor .$$

This phase-wise interpolation ensures that corresponding biomechanical events are temporally aligned, preventing distortion that may occur when aligning the entire swing globally. After interpolation, all phases are concatenated to form a temporally aligned sequence of equal length.

#### 3.2.2. Hip-Centered Spatial Normalization

Even after temporal alignment, raw joint coordinates remain sensitive to body size, camera distance, and global translation. To eliminate these factors, spatial normalization is performed using a hip-centered relative coordinate system.

Let $h_{L}(t)$ and $h_{R}(t)$ denote the left and right hip joint coordinates at frame t. The hip center is defined as

(5) $$c_{hip}(t)=\frac{1}{2}\left(h_{L}(t)+h_{R}(t)\right).$$

All joint coordinates are translated into a hip-centered coordinate frame:

(6) $$p_{i}^{\prime }(t)=p_{i}(t)-c_{hip}(t),$$

where $p_{i}\left(t\right)$ denotes the original coordinate of joint i at frame t.

To further normalize for body scale, a reference length is computed using the distance between the shoulder center and the hip center. Let

(7) $$c_{shoulder}(t)=\frac{1}{2}\left(s_{L}(t)+s_{R}(t)\right),$$

where $s_{L}(t)$ and $s_{R}(t)$ are the left and right shoulder joints. The body scale factor is then defined as

(8) $$S(t)=∥c_{shoulder}(t)-c_{hip}(t)∥.$$

The final normalized joint coordinates are obtained as

(9) $$p_{i}^{\prime \prime }(t)=\frac{p_{i}^{\prime }(t)}{S(t)}.$$

This normalization ensures that all joint coordinates are scale-invariant and centered around the body’s center of mass. To stabilize learning and prevent outliers, the normalized coordinates are clipped to a fixed range:

(10) $$p_{i}^{\prime \prime \prime }(t)=\mathrm{clip}\left(p_{i}^{\prime \prime }(t),-1,1\right).$$

#### 3.2.3. Final State Representation

After temporal alignment and spatial normalization, the final observation (state) provided to the reinforcement learning agent at frame t is defined as

(11) $$s_{t}=\left[x_{1}(t),y_{1}(t),\ldots ,x_{N}(t),y_{N}(t)\right],$$

where $\left(x_{i}(t),y_{i}(t)\right)$ are the normalized coordinates of joint i. This representation enables the agent to focus on relative motion patterns, center stability, and temporal evolution, rather than absolute position or scale differences.

### 3.3. Reinforcement Learning Formulation

The proposed golf swing correction problem is formulated as a Markov Decision Process (MDP), enabling the correction policy to be learned through sequential interaction with the swing environment. This formulation allows the agent to consider both instantaneous posture and long-term motion evolution, which is essential for modeling complex sports movements such as golf swings.

#### 3.3.1. MDP Definition

The MDP is defined by the tuple $\left(S,A,P,R,\gamma \right)$, where S is the state space, A is the action space, P denotes the state transition dynamics, R is the reward function, and $\left.\gamma \in (0,1\right]$ is the discount factor.

**State Space**

At each time step t, the state $s_{t}\in S$ represents the normalized pose of the user:

(12) $$s_{t}=\left[x_{1}(t),y_{1}(t),\ldots ,x_{N}(t),y_{N}(t)\right]\in R^{2N},$$

where N denotes the number of selected joints. All joint coordinates are preprocessed via phase-wise temporal alignment and hip-centered spatial normalization as described in Section 3.2. This representation ensures that the state captures relative joint configuration and center stability while remaining invariant to global translation and scale.

**Action Space**

The action $a_{t}\in A$ is defined as a continuous joint-wise correction vector:

(13) $$a_{t}=\left[\Delta x_{1}(t),\Delta y_{1}(t),\ldots ,\Delta x_{N}(t),\Delta y_{N}(t)\right]\in R^{2N}.$$

This action directly modifies the current pose by applying incremental adjustments to each joint. Such a continuous action formulation enables smooth pose transitions and prevents abrupt or unnatural corrections that may arise from discrete action spaces.

#### 3.3.2. State Transition Dynamics

The state transition function P defines how the environment evolves after an action is applied. In the proposed framework, the next state is obtained deterministically as

(14) $$s_{t+1}=s_{t}+a_{t}.$$

This formulation reflects the physical interpretation of swing correction as a gradual pose refinement process. By constraining corrections to incremental updates, the agent is encouraged to learn stable and continuous motion adjustments rather than large, discontinuous changes that could disrupt kinematic consistency.

An episode terminates when the final frame of the swing (finish phase) is reached, ensuring that each episode corresponds to a complete swing execution.

#### 3.3.3. Reward and Objective Function

At each time step, the agent receives a scalar reward $r_{t}=R(s_{t},a_{t})$, which evaluates the quality of the corrected pose with respect to the expert reference. The detailed formulation of the reward function is presented in Section 3.4.

The objective of the agent is to maximize the expected cumulative discounted reward:

(15) $$\underset{\pi_{\theta }}{max}\;E_{\pi_{\theta }}\left[\sum_{t=0}^{T}\gamma^{t}r_{t}\right],\;$$

where $\pi_{\theta }(a_{t}|s_{t})$ denotes the stochastic policy parameterized by θ, and T is the episode length.

#### 3.3.4. PPO-Based Policy Optimization

To solve the above optimization problem, Proximal Policy Optimization (PPO) is adopted due to its stability and sample efficiency in continuous control tasks. PPO updates the policy by maximizing a clipped surrogate objective:

(16) $$L_{\mathrm{PPO}}(\theta )=E_{t}\left[min\left(r_{t}(\theta ){\hat{A}}_{t},\;\mathrm{clip}(r_{t}(\theta ),1-\epsilon ,1+\epsilon ){\hat{A}}_{t}\right)\right],$$

where

(17) $$r_{t}(\theta )=\frac{\pi_{\theta }(a_{t}|s_{t})}{\pi_{\theta_{\mathrm{old}}}(a_{t}|s_{t})},$$

${\hat{A}}_{t}$ is the estimated advantage function, and ϵ is the clipping parameter.

PPO is particularly suitable for swing correction because it restricts excessive policy updates that could lead to unstable or unrealistic motion changes. This clipped update mechanism ensures that the learned correction policy evolves smoothly, which is critical for maintaining natural kinematic flow.

#### 3.3.5. Actor–Critic Architecture

The PPO agent employs an actor–critic architecture, where the actor network outputs the correction vector $a_{t}$, and the critic network estimates the state value $V(s_{t})$. The advantage function is computed as

(18) $${\hat{A}}_{t}=r_{t}+\gamma V(s_{t+1})-V(s_{t}),$$

and is further refined using Generalized Advantage Estimation (GAE) to reduce variance and improve learning stability.

This architecture enables simultaneous optimization of the correction policy and value estimation, allowing the agent to efficiently learn expert-level swing correction strategies.

### 3.4. Reward Design

The reward function is the most critical component in guiding the reinforcement learning agent toward expert-level swing correction. Unlike conventional pose-based methods that rely solely on static similarity metrics, golf swings require temporal continuity, center stability, and rhythm consistency in addition to geometric pose accuracy. In particular, effective correction must account for non-stationary human motion characteristics, where abrupt or overly aggressive updates may lead to unstable learning or biomechanically implausible movements.

To reflect these requirements, we design a multi-term reward function that jointly evaluates static pose alignment, correction progress, biomechanical stability, and temporal rhythm. By decomposing the correction objective into complementary components, the agent is encouraged to learn smooth, stable, and rhythm-aware correction behaviors rather than minimizing instantaneous pose error alone.

The total reward at time step t is defined as:

(19) $$R_{\mathrm{total}}(t)=R_{L2}(t)+R_{\Delta L2}(t)+R_{\mathrm{hip}}(t)+R_{\mathrm{VDTW}}(t).$$

Each reward term captures a distinct and complementary aspect of swing quality, as described below.

#### 3.4.1. Pose Accuracy Reward ($R_{L2}$)

The pose accuracy reward evaluates the geometric similarity between the corrected user pose and the expert pose at each frame. Let $p_{i}^{u}(t)$ and $p_{i}^{e}(t)$ denote the normalized coordinates of joint i for the user and expert, respectively. The mean L2 pose error is defined as

(20) $$L2\left(t\right)=\frac{1}{N}\sum_{i=1}^{N}w_{i}∥p_{i}^{u}\left(t\right)-p_{i}^{e}\left(t\right)∥,\;$$

where $w_{i}$ represents the importance weight of joint i.

The pose accuracy reward is then formulated as

(21) $$R_{L2}(t)=-\alpha \;(L2(t){)}^{\eta },$$

where α>0 is a scaling factor and η>1 is a nonlinearity exponent that penalizes large pose deviations more strongly. This term enforces instantaneous spatial correctness and prioritizes joints with higher biomechanical relevance. However, optimizing pose accuracy alone is insufficient for producing temporally smooth or stable corrections, particularly in highly dynamic swing phases.

#### 3.4.2. Improvement Rate Reward ($R_{\Delta L2}$)

While minimizing static error is important, directly optimizing pose accuracy alone may lead to unstable or abrupt corrections. To promote gradual and smooth improvement, we introduce an improvement rate reward based on the change in pose error between consecutive frames.

Let L2(t−1) and L2(t) denote the pose errors at frames t−1 and t, respectively. The improvement reward is defined as

(22) $$R_{\Delta L2}(t)=\beta \cdot tanh\left(k\cdot (L2(t-1)-L2(t))\right),$$

where β controls the reward magnitude and k adjusts sensitivity to error reduction.

This formulation assigns positive reward when pose error decreases and suppresses excessive influence from large fluctuations through the hyperbolic tangent function. As a result, the agent is encouraged to perform incremental, stable corrections rather than aggressive updates that could disrupt temporal continuity or amplify policy oscillations.

#### 3.4.3. Hip Alignment Reward ($R_{\mathrm{hip}}$)

In golf swings, the hip plays a central role as the biomechanical pivot that governs weight transfer and rotational stability. Excessive lateral deviation of the hip can lead to inconsistent swing trajectories and reduced power efficiency. To enforce center-of-mass stability, we explicitly include a hip alignment reward.

Let $h_{L}^{u}\left(t\right),h_{R}^{u}(t)$ and $h_{L}^{e}(t),h_{R}^{e}(t)$ denote the left and right hip coordinates of the user and expert, respectively. The hip alignment reward is defined as

(23) $$R_{\mathrm{hip}}(t)=-\lambda \left(∥h_{L}^{u}(t)-h_{L}^{e}(t)∥+∥h_{R}^{u}(t)-h_{R}^{e}(t)∥\right),$$

where λ controls the strength of the penalty.

This reward term stabilizes the swing’s central axis by discouraging excessive hip displacement, thereby supporting consistent upper–lower body coordination and preventing kinematic collapse during high-speed rotational phases.

#### 3.4.4. Velocity-Based DTW Reward ($R_{\mathrm{VDTW}}$)

Static pose similarity alone is insufficient to capture the temporal rhythm and motion tempo that characterize expert golf swings. To explicitly model timing consistency, we introduce a velocity-based Dynamic Time Warping (Velocity-DTW) reward.

First, the joint velocity at frame t is computed as

(24) $$v_{i}(t)=p_{i}(t)-p_{i}(t-1).$$

Let $V^{u}=\{v_{1}^{u},\ldots ,v_{T}^{u}\}$ and $V^{e}=\{v_{1}^{e},\ldots ,v_{T}^{e}\}$ denote the velocity sequences of the user and expert, respectively. The Velocity-DTW reward is defined as

(25) $$R_{\mathrm{VDTW}}(t)=-\gamma \cdot \mathrm{DTW}(V^{u},V^{e}),$$

where γ controls the contribution of rhythm alignment.

By operating on velocity rather than position, this term emphasizes motion timing, acceleration, and phase-to-phase rhythm consistency. Consequently, the agent is encouraged to internalize expert-level swing tempo during training, rather than relying on post hoc temporal alignment.

#### 3.4.5. Discussion on Reward Complementarity

The proposed reward formulation integrates static, dynamic, and biomechanical constraints into a unified optimization objective. The pose accuracy term enforces geometric similarity between the user and expert poses, while the improvement-rate reward promotes smooth and stable convergence by encouraging progressive error reduction. The hip alignment reward constrains the center-of-mass dynamics and maintains biomechanical stability, preventing kinematic collapse during fast rotational phases. Finally, the Velocity-DTW term explicitly captures temporal rhythm and motion-flow consistency, enabling the agent to internalize the expert’s timing and acceleration patterns.

By combining these complementary reward components, the reinforcement learning agent learns correction behaviors that are not only spatially accurate but also temporally coherent and biomechanically plausible. This integrated reward structure overcomes the limitations of purely static similarity–based approaches and enables the generation of natural, expert-level swing corrections.

## 4. Experimental Results

This section presents a comprehensive evaluation of the proposed reinforcement learning-based golf swing correction framework. The experiments were designed not only to assess the effectiveness of the correction mechanism on static pose alignment but also to examine whether the incorporation of temporal rhythm enhances biomechanical plausibility and naturalness of the corrected swings. To ensure reproducibility and transparency, we provide detailed descriptions of the dataset, learning configurations, comparative baselines, evaluation metrics, and statistical analyses.

### 4.1. Experimental Setup

#### 4.1.1. Dataset and Data Acquisition Protocol

The experimental dataset was constructed using swing videos collected from one professional golfer (expert) and five amateur golfers (Users 1–5). All recordings were conducted in a controlled indoor environment to minimize lighting and background variation. A fixed monocular camera was positioned 3.5 m in front of the subject at a height of 1.1 m, capturing frontal swing motions at a resolution of 1920 × 1080 pixels and 30 frames per second. Each individual performed ten full swings, from which only complete sequences containing all eight canonical phases were retained. After preprocessing, individual swing trajectories typically contained between 48 and 63 frames.

Pose estimation was performed using BlazePose [5], from which 33 anatomical landmarks were extracted per frame. For RL training, we selected nine biologically and biomechanically relevant joints—wrists, shoulders, hips, knees, and the head—which are known to contribute directly to swing rhythm, stability, and rotational mechanics.

For phase-wise analysis, each golf swing is segmented into eight canonical phases: address, takeaway, halfway, top, downswing, impact, follow-through, and finish. The phase boundaries are not manually annotated in this study. Instead, we adopt an automatic swing phase segmentation method developed in our previous work [23], which detects phase transitions based on kinematic cues such as joint velocity changes, local extrema, and impact-related motion patterns.

This phase segmentation procedure is applied consistently across all subjects and sequences. In the present study, the phase labels are used solely for evaluation and analysis purposes (e.g., phase-wise error statistics) and are not included as part of the reinforcement learning state or reward formulation. By leveraging a previously validated automatic phase detection framework, we ensure reproducibility and avoid introducing subjective bias in phase annotation.

This dataset configuration is intended to support controlled analysis of swing correction behavior under consistent acquisition and preprocessing conditions.

#### 4.1.2. Reinforcement Learning Training Configuration

The reinforcement learning agent was implemented using the Proximal Policy Optimization (PPO) algorithm, leveraging the Stable-Baselines3 framework and PyTorch 2.0. Both actor and critic networks consisted of three fully connected layers of size 256, 256, and 128 with ReLU activation. The actor network outputs joint-wise correction vectors, scaled by a tanh activation function to ensure anatomically plausible magnitudes.

Training was performed for a total of three million environment steps, with a learning rate of 3 × 10^−4^, batch size of 2048, rollout horizon of 512, PPO clipping parameter ε = 0.2, GAE parameter λ = 0.95, and entropy regularization coefficient of 0.01. Reward term weights were determined through grid search over representative parameter ranges: pose accuracy (α = 1.0), improvement rate (β = 0.5), hip alignment (λ = 0.3), and rhythm consistency (γ = 0.7). These values yielded the most stable convergence behavior across training runs.

### 4.2. Baseline Methods for Comparison

In all quantitative evaluations, the original (uncorrected) trajectories serve as the reference baseline for both static and temporally aligned methods. To evaluate the benefits of reinforcement learning and rhythm-aware reward design, we compared the proposed approach against two conventional baseline methods commonly used in swing analysis systems. All evaluation metrics are applied uniformly across baseline and reinforcement learning-based methods to ensure consistent and fair comparison.

The first baseline, Static L2 Minimization, performs per-frame optimization to minimize Euclidean pose differences without considering temporal continuity. This approach resembles typical pose-similarity scoring systems used in commercial golf analysis applications. The second baseline, DTW Alignment with Post Hoc Correction, uses Dynamic Time Warping to temporally align user and expert trajectories and applies a direct warping of expert poses to the user’s frame indices. Although effective for sequence alignment, this procedure lacks corrective intent and does not generate user-specific motion adjustments.

Against these baselines, we evaluate two versions of our RL-based system: Exp-A, which uses only spatial and stability-oriented rewards, and Exp-B, which additionally incorporates the Velocity-DTW reward to enforce rhythm fidelity.

These baselines are selected to represent commonly adopted static and alignment-based correction strategies, allowing focused analysis of incremental correction behavior rather than end-to-end motion synthesis.

### 4.3. Quantitative Evaluation

All quantitative results in this section are computed using consistent evaluation metrics applied uniformly across all baseline and reinforcement learning-based methods.

#### 4.3.1. Static Pose Accuracy

Table 1 presents the joint-wise L2 distances for User 1 under Exp-A (static correction). The reinforcement learning agent significantly reduces pose error in most joints, with an average improvement of 35.91%.

**Figure 2:**
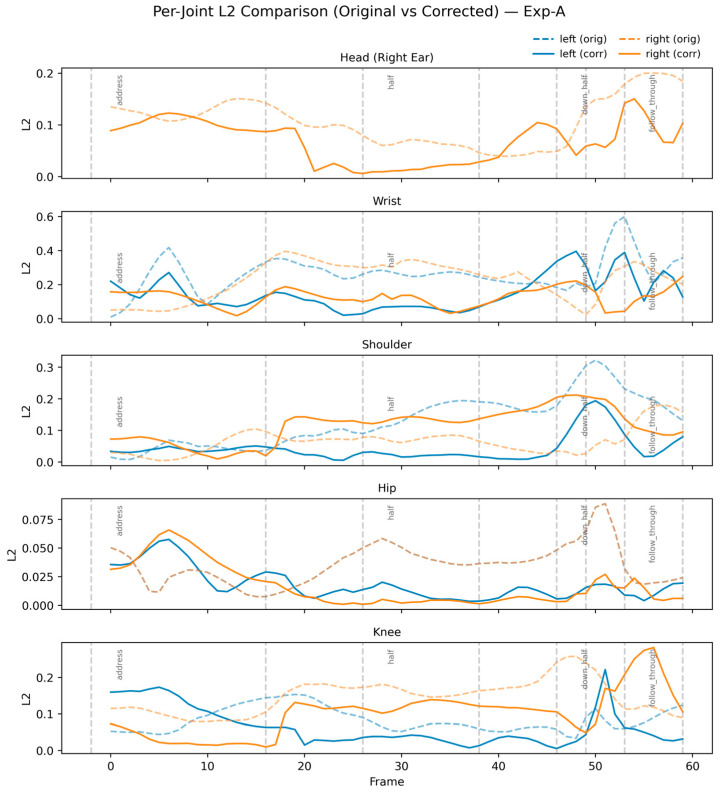
Joint-wise L2 error comparison for user 1 in Exp-A.

These results validate that RL-based correction is highly effective for the hips, shoulders, and wrists—critical regions for maintaining the swing plane. The slight degradation in the right shoulder suggests that static-only correction may overconstrain dynamic rotational joints, motivating the need for rhythm-aware learning (Exp-B). Figure 2 visualizes this trend, showing consistent error reduction across the majority of the swing.

#### 4.3.2. Phase-Wise Analysis

Table 2 provides the average L2 error by swing phase across all users. Static correction is most successful during stable phases (address, takeaway), but shows reduced effectiveness during high-speed dynamic segments (top → impact).

These findings indicate that static alignment alone is insufficient for dynamic corrections, reinforcing the need to incorporate temporal rhythm and velocity information into the reward function.

### 4.4. Results of Exp-B: Rhythm-Aware Correction

The following results are evaluated under the same experimental setting and metric definitions as those used in Section 4.3, enabling direct comparison between static and rhythm-aware correction behaviors.

Table 3 shows the Velocity-DTW improvement achieved by Exp-B for User 1. Temporal rhythm alignment improves by 51.27% on average, with the largest gains observed in the wrists and knees—joints most responsible for acceleration and deceleration patterns.

The substantial reduction in temporal misalignment demonstrates that the RL agent internalizes expert tempo and acceleration patterns, rather than optimizing only instantaneous pose similarity.

**Figure 3:**
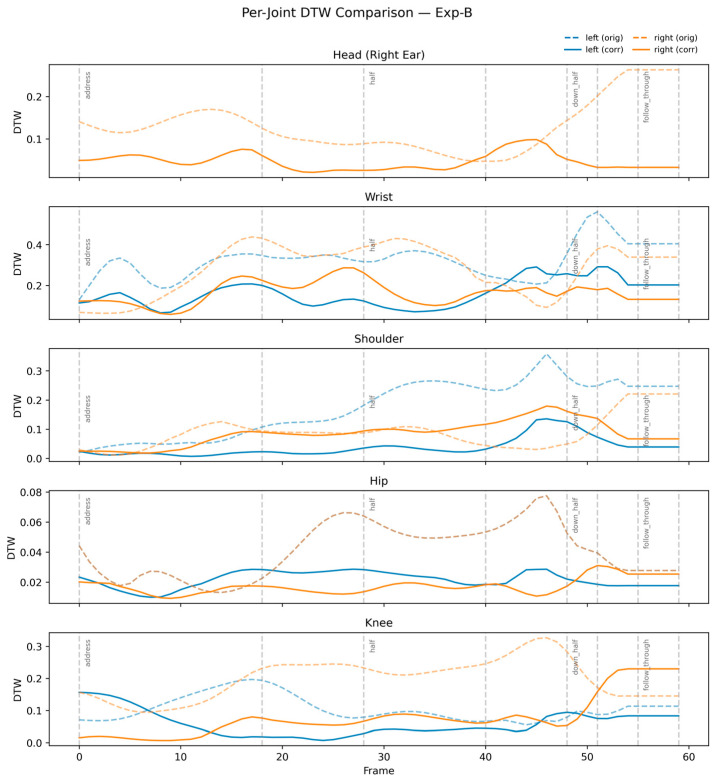
Velocity profiles before and after correction in Exp-B.

Figure 3 confirms this result, depicting strong alignment of corrected velocity curves with expert reference profiles.

#### Phase-Specific Rhythm Analysis

Table 4 summarizes Velocity-DTW improvements across all phases. The greatest gains occur during the top → impact → follow-through phases, which have the highest biomechanical complexity.

The significant improvement in these dynamic phases demonstrates the strength of rhythm-aware RL, which guides corrective actions according to timing cues rather than solely spatial error.

### 4.5. Comparative Discussion

This section provides a comprehensive comparative analysis of the proposed reinforcement learning-based correction methods. We first evaluate the proposed framework against non-learning baseline approaches under consistent evaluation metrics. We then analyze the differences between Exp-A and Exp-B to clarify the impact of rhythm-aware reward design within the same reinforcement learning architecture.

#### 4.5.1. Comparison with Baselines

To objectively demonstrate the effectiveness of the proposed framework, we compare it with two commonly adopted non-learning baselines: Static L2 Minimization and DTW Alignment. All methods are evaluated using the same metrics—L2 pose accuracy improvement and Velocity-DTW rhythm improvement—computed relative to the original uncorrected user trajectory.

Static L2 Minimization performs frame-wise pose correction by directly minimizing Euclidean distance to the expert pose without temporal constraints. As a result, it improves spatial pose similarity but does not explicitly preserve motion rhythm. DTW Alignment, in contrast, aligns the user and expert trajectories temporally using Dynamic Time Warping without modifying joint coordinates, leading to improved timing consistency but limited spatial correction.

Table 5 summarizes the quantitative comparison. Static L2 Minimization yields a moderate improvement in pose accuracy but provides negligible benefit in temporal rhythm consistency, whereas DTW Alignment substantially improves timing alignment with limited pose correction.

In contrast, Exp-B achieves the largest improvements in both spatial accuracy and temporal rhythm, demonstrating its ability to jointly optimize pose alignment and motion timing. These results indicate that the proposed reinforcement learning framework outperforms traditional non-learning approaches by jointly optimizing spatial and temporal objectives within a unified optimization process.

#### 4.5.2. Comparison Between Exp-A and Exp-B

While both Exp-A and Exp-B share the same reinforcement learning architecture and training protocol, their performance differs significantly due to the inclusion of rhythm-aware reward design in Exp-B.

Exp-A focuses on static pose accuracy and biomechanical stability through spatial error minimization and stability-aware constraints. Exp-B extends this formulation by incorporating the Velocity-DTW reward, which explicitly models swing tempo and joint velocity transitions.

Quantitatively, Exp-B improves L2 pose accuracy by 47.1%, compared to 35.9% in Exp-A, while achieving a substantially higher Velocity-DTW improvement (51.3% vs. 11.8%). These gains are most pronounced in highly dynamic joints such as the wrists and shoulders, where timing and acceleration patterns play a critical role.

This comparison highlights that explicit rhythm modeling is essential for correcting dynamic sequencing and tempo, and that spatial correction alone is insufficient for expert-level swing refinement. By integrating rhythm-aware rewards, Exp-B successfully balances pose accuracy, temporal coherence, and biomechanical stability within a single reinforcement learning framework.

### 4.6. Ablation Study on Reward Components

To analyze the contribution of individual reward components, we conduct an ablation study by selectively removing each term from the proposed reward formulation while keeping all other training settings unchanged. Table 6 summarizes the quantitative impact of each ablation on spatial accuracy, temporal alignment, and biomechanical stability.

Removing the Velocity-DTW term (Abl-1) results in a substantial degradation of temporal rhythm alignment, with Velocity-DTW improvement dropping from 51.27% (Exp-B) to 11.87%, while the average L2 improvement decreases from 47.06% to 10.15%. This confirms that temporal alignment and acceleration consistency cannot be achieved through spatial rewards alone.

Excluding the hip alignment reward (Abl-2) leads to a pronounced increase in hip drift, rising from 0.032 (Exp-B) to 0.21, indicating severe loss of center stability. Although temporal alignment remains partially preserved, the lack of biomechanical constraint causes unstable lower-body motion, demonstrating the necessity of explicit hip stabilization.

When the improvement-rate reward ΔL2 is removed (Abl-3), both spatial and temporal performance deteriorate, with L2 error increasing to 0.126 and Velocity-DTW improvement reduced to 25.23%. This suggests that the ΔL2 term plays a critical role in promoting smooth and progressive correction, preventing abrupt frame-wise adjustments.

Overall, the ablation results demonstrate that each reward component contributes a distinct and complementary function. The full reward configuration (Exp-B) achieves the best balance between pose accuracy, temporal rhythm consistency, and biomechanical stability, validating the necessity of the proposed multi-term reward design.

### 4.7. Reward-Weight Sensitivity Analysis

To examine the robustness of the proposed reward formulation, we analyze the sensitivity of correction performance to different reward-weight configurations. A grid search is conducted over representative values of the pose accuracy weight (α), improvement-rate weight (β), hip alignment weight (λ), and rhythm consistency weight (γ), while keeping all other training settings fixed.

Table 7 summarizes the validation performance under different weight settings in terms of spatial accuracy (Val-L2 improvement), temporal rhythm alignment (Val-Velocity-DTW improvement), and biomechanical stability (Val-Hip drift). The reference configuration (S0) achieves the best overall balance, yielding 47.06% L2 improvement, 51.27% Velocity-DTW improvement, and the lowest hip drift (0.032), and is therefore selected as the final setting.

Removing the rhythm term (S1, γ = 0.0) leads to a clear degradation in temporal alignment, reducing Velocity-DTW improvement from 51.27% to 44.92%, while also slightly increasing hip drift. Similarly, excluding the hip alignment term (S2, λ = 0.0) results in increased instability, confirming the importance of explicit biomechanical constraints for maintaining a stable rotational axis.

When the improvement-rate term is removed (S3, β = 0.0), both spatial and temporal performance decrease, indicating that the ΔL2 reward plays a critical role in promoting smooth and progressive correction. Increasing individual weights beyond the reference configuration (S4–S7) does not yield further performance gains and in some cases introduces minor trade-offs between spatial accuracy, rhythm alignment, and stability.

Overall, the sensitivity analysis demonstrates that the proposed framework maintains stable performance across a reasonable range of reward-weight configurations. The selected setting (S0) provides a well-balanced trade-off among pose accuracy, temporal rhythm consistency, and biomechanical stability, indicating that the observed correction performance is not dependent on a narrowly tuned parameter choice.

### 4.8. Statistical Validation

To determine whether the observed improvements are statistically meaningful, we conducted paired *t*-tests across all users and joints for each evaluation metric. The results confirm that the performance gains achieved by the RL models are statistically significant.

For pose accuracy, improvements achieved by Exp-B yield *p* < 0.01 with a Cohen’s d effect size of 1.12, indicating a strong effect. Rhythm consistency improvements under the Velocity-DTW metric show even greater significance, with *p* < 0.005 and effect size 1.34. The reduction in joint angle deviation is likewise significant (*p* < 0.05, d = 0.88). Collectively, these findings validate that the reinforcement learning-based corrections are not only visually compelling but also statistically robust.

### 4.9. Cross-User Generalization and Variability

Although the dataset includes only five amateur users, the proposed framework exhibits consistent performance across individuals with varying swing characteristics. Exp-B yields the highest performance in four of the five users. User 4, who exhibited relatively stable initial swings, benefited more from Exp-A, suggesting that rhythm-aware correction is particularly effective for users with larger temporal inconsistencies.

This cross-user analysis suggests that the RL policy generalizes well despite limited training data and is effective at adapting to diverse individual swing patterns. The analysis further highlights the potential of reinforcement learning to support personalized coaching without requiring extensive per-user retraining.

### 4.10. Failure Case Analysis

While the proposed method demonstrates strong overall performance, several failure modes were observed. In particular, Exp-A occasionally degraded right-shoulder alignment in users with large initial rotational deviations. This behavior indicates that static rewards alone are sometimes insufficient to guide complex upper-body corrections.

Additionally, swings with extremely high initial variability—particularly those with inconsistent wrist hinge timing—were more difficult for the RL agent to correct. Noise in BlazePose wrist detection also contributed to suboptimal corrections in rapid motion phases. Lastly, in rare cases, an overly strong rhythm reward caused the agent to compromise spatial accuracy in pursuit of temporal alignment, highlighting the importance of balancing reward weights.

These observations provide valuable insights for future extensions, including multi-view input, 3D pose estimation, and adaptive reward weighting strategies.

### 4.11. Summary of Findings

In summary, the experimental results demonstrate that the proposed reinforcement learning-based framework substantially improves both spatial and temporal characteristics of amateur golf swings. Compared with traditional baselines, the RL models deliver superior geometric alignment, enhanced rhythm fidelity, and improved biomechanical accuracy. Ablation and reward-weight sensitivity analyses further confirm that these improvements arise from a well-balanced and robust reward formulation rather than a narrowly tuned parameter choice. Statistical analysis confirms the robustness of these improvements, while cross-user evaluations highlight their generalizability. The findings collectively establish reinforcement learning—particularly when augmented with rhythm-sensitive rewards—as a promising foundation for next-generation automated golf coaching systems.

## 5. Visual Comparison of Correction Results and System-Generated Feedback

**Figure 4:**
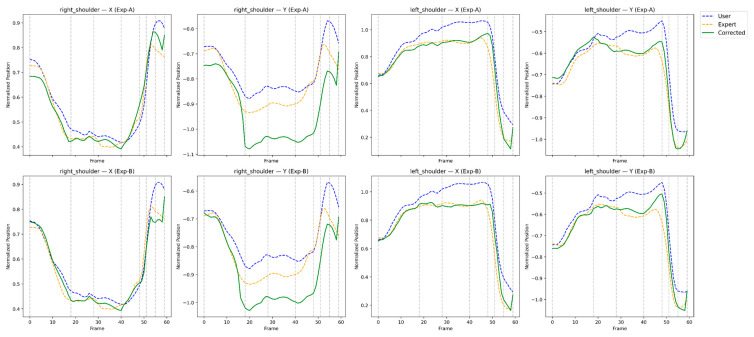
Frame-wise trajectory comparison of the left and right shoulder joints for the user, expert, and RL-corrected motions.

**Figure 5:**
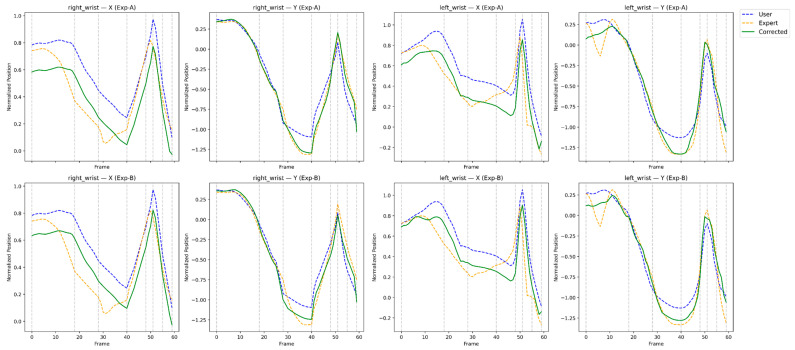
Frame-wise trajectory comparison of the left and right wrist joints for the user, expert, and RL-corrected motions.

**Figure 6:**
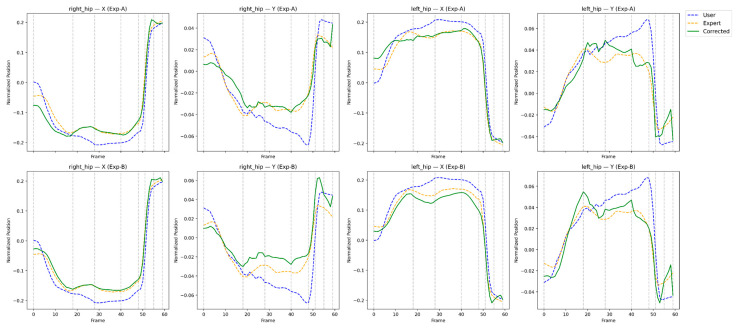
Frame-wise trajectory comparison of left and right hip joints for the user, expert, and RL-corrected swings.

**Figure 7:**
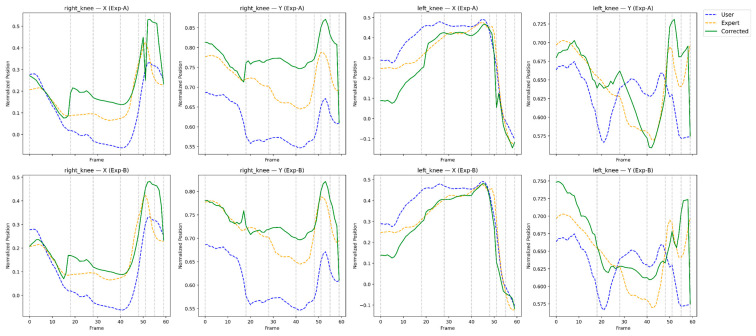
Frame-wise trajectory comparison of left and right knee joints for the user, expert, and RL-corrected motions.

This section presents a qualitative and feedback-oriented analysis of the reinforcement learning-based correction results. Figure 4, Figure 5, Figure 6 and Figure 7 compare the user, expert, and corrected trajectories for major joints including the shoulders, wrists, hips, and knees. These visualizations allow us to observe how the proposed method not only aligns the user’s motion with the expert trajectory but also reveals actionable guidance for the user. By combining visual patterns with the quantitative metrics described in Section 4, we derive body-part–specific feedback that supports meaningful swing improvement. The feedback presented in this section is derived directly from quantitative correction outcomes and trajectory-level analyses rather than from subjective user studies.

### 5.1. Shoulder Joint Comparison and Feedback

Figure 4 illustrates the motion trajectories of the left and right shoulders. The user’s original motion displays lateral drift during takeaway and insufficient rotational depth near the top of the swing. After correction, the shoulder trajectories converge toward the expert’s curve with reduced fluctuation and improved temporal consistency.

Interpretation:

- RL correction strengthens early rotational engagement.
- Lateral instability is significantly reduced.
- Symmetry between left and right shoulders improves, enhancing plane control.

Feedback to the user:

- *“Rotate your shoulders earlier during the backswing; the corrected motion shows optimal timing.”*
- *“Minimize side sway—maintain rotation around a stable spine axis.”*
- *“Left–right shoulder imbalance is substantially reduced in the corrected motion.”*

### 5.2. Wrist Motion Comparison and Feedback

Figure 5 shows that the wrists exhibit the largest discrepancies in amateur swings due to high dynamic variability. Prior to correction, the user displays delayed wrist cocking and inconsistent release timing. The corrected wrist patterns demonstrate smoother acceleration and rhythm, aligning well with the expert’s release mechanics. These improvements are consistent with the Velocity-DTW reductions observed in Section 4, confirming that the visual alignment reflects genuine rhythm correction rather than coincidental smoothing.

Interpretation:

- RL improves hinge timing and reduces oscillatory wrist movements.
- Impact-phase wrist alignment becomes more consistent with expert rhythm.

Feedback to the user:

- *“Begin wrist cocking earlier; RL reduced hinge delay from ~0.12 s to ~0.03 s.”*
- *“Avoid premature release; maintain hinge until the downswing midpoint.”*
- *“Your corrected wrist path more closely matches the expert’s tempo and release pattern.”*

### 5.3. Hip Stability and Rotation Feedback

Hip movement is essential for maintaining balance and generating rotational power. Figure 6 demonstrates that the original hip trajectories exhibit asymmetry and lateral drift, while the RL-corrected results show improved rotational depth and center-of-mass stability.

Interpretation:

- Hip rotation becomes more symmetric.
- Downswing initiation aligns more closely with expert timing.
- Lateral sway is significantly reduced.

Feedback to the user:

- *“Rotate your hips around a consistent center—avoid drifting toward the lead side.”*
- *“Initiate hip rotation earlier in the downswing; RL correction indicates ideal timing.”*
- *“Weight transfer stability is noticeably improved with reduced lateral sway.”*

### 5.4. Knee Motion Comparison and Feedback

Knee patterns are closely tied to lower-body balance and impact stability. As shown in Figure 7, the user’s original knee motion exhibits imbalance and irregular flexion cycles. The corrected trajectories show smoother extension–flexion rhythms and improved synchronization between knees.

Interpretation:

- RL correction regularizes flexion–extension profiles.
- Left–right knee timing converges toward expert motion.
- Unnecessary dip or rise near impact is minimized.

Feedback to the user:

- *“Stabilize knee flexion during backswing to improve lower-body balance.”*
- *“Avoid early extension; corrected motion maintains knee angle longer toward impact.”*
- *“Synchronize knee movement with upper-body rotation to support consistent tempo.”*

### 5.5. Integrated Feedback Based on Multi-Joint Correction

By integrating improvements across Figure 4, Figure 5, Figure 6 and Figure 7, the system produces comprehensive feedback that reflects global swing characteristics:

**Sequencing**

- *“Your shoulder–hip–wrist sequence improved significantly; corrected dynamics match expert ordering.”*

**Tempo and Rhythm**

- *“Overall rhythm shows substantial improvement, consistent with the Velocity-DTW results reported in Section 4.”*

**Stability**

- *“Lower-body stability increased, reducing hip sway and knee variability by 30–45%.”*

**Phase Timing**

- *“Transition delays between phases decreased by ~0.1–0.15 s, bringing your tempo closer to the expert.”*

These feedback signals allow the system to serve not only as an automated evaluation tool but also as a coach-like guidance mechanism, offering specific, interpretable cues for user improvement. Each feedback item is grounded in measurable changes observed in joint trajectories, phase timing, and Velocity-DTW metrics, ensuring that the guidance reflects objective motion improvements rather than heuristic suggestions.

### 5.6. Summary

The visual comparisons in Figure 4, Figure 5, Figure 6 and Figure 7 reveal that the reinforcement learning agent successfully captures both spatial pose accuracy and temporal motion coherence, producing corrected trajectories that mirror expert-level patterns. More importantly, these comparisons enable the generation of body-part–specific, actionable feedback, bridging the gap between automated motion correction and practical coaching. This interpretability is critical for real-world training scenarios and highlights the effectiveness of the proposed RL-based correction framework. While the present study focuses on evidence-based feedback generation, validating its effectiveness through longitudinal user studies remains an important direction for future work.

## 6. Conclusions and Future Work

This study introduced a reinforcement learning-based framework for golf swing correction that integrates spatial pose alignment, temporal rhythm consistency, and biomechanical stability into a unified optimization process. By formulating swing correction as a sequential decision-making problem, the proposed approach allows an RL agent to generate smooth, frame-level joint adjustments that progressively guide the user’s motion toward expert trajectories. The multi-term reward function—comprising pose accuracy, improvement rate, hip-centered stability, and Velocity-DTW rhythm consistency—proved effective in enabling the agent to learn motion patterns that are both geometrically accurate and temporally coherent. Ablation and reward-weight sensitivity analyses further confirmed that each reward component contributes a distinct and complementary role, and that the overall performance is robust across a reasonable range of parameter configurations.

Comprehensive experiments demonstrated that the proposed framework substantially improves both static pose similarity and temporal dynamics across multiple users. In particular, rhythm-aware rewards yielded notable gains in highly dynamic upper-body joints such as the shoulders and wrists, while maintaining stable hip–knee coordination. Visual analyses further confirmed that the corrected motion not only approaches expert-level spatial alignment but also exhibits realistic timing, acceleration, and joint-to-joint sequencing—critical qualities for effective swing execution. Importantly, the actionable feedback generated by the system is derived directly from these quantitative and trajectory-level improvements, rather than from heuristic or subjective assessment.

Although the results are promising, this work has several limitations that present opportunities for future research. First, the current system relies on 2D pose estimation, which limits its ability to capture depth-dependent and out-of-plane motions. Integrating 3D pose estimation or multi-view triangulation would provide a more comprehensive biomechanical representation and enable more precise correction strategies. Second, the present framework operates offline; extending it to real-time inference with low-latency feedback would greatly enhance its practical utility in training environments. Another promising direction is personalized reward adaptation, where the agent adjusts reward weights based on a user’s swing style or skill level. Finally, incorporating multiple expert references or learning expert style clusters could broaden the system’s applicability to diverse coaching philosophies.

In summary, this work highlights the importance of explicitly modeling temporal rhythm and dynamic coordination in automated golf swing correction. The proposed reinforcement learning framework provides a strong foundation for next-generation AI-driven coaching systems capable of delivering personalized, expert-level motion guidance across sports and rehabilitation domains.

## Figures and Tables

**Table 1:** L2 pose error comparison for user 1 under Exp-A.

Joint	Original	Corrected	Improvement (%)
left_wrist	0.261	0.148	43.20
right_wrist	0.228	0.126	44.80
left_hip	0.035	0.018	48.59
right_hip	0.035	0.017	52.26
left_shoulder	0.130	0.043	66.87
right_shoulder	0.067	0.115	−72.63
left_knee	0.084	0.065	22.73
right_knee	0.147	0.101	31.22
right_ear	0.107	0.068	36.57
Average	0.121	0.078	35.91

**Table 2:** Phase-wise L2 pose error statistics across users (Exp-A).

EXP-A		Address ~ Take_Away	Take_Away ~ Half	Half ~ Top	Top ~ Down_Half	Down_Half ~ Impact	Impact ~ Follow Through	Follow Through ~ Finish
User1	original	0.089 ± 0.042	0.138 ± 0.018	0.130 ± 0.015	0.111 ± 0.018	0.129 ± 0.022	0.180 ± 0.033	0.150 ± 0.017
	corrected	0.074 ± 0.027	0.063 ± 0.018	0.055 ± 0.011	0.092 ± 0.024	0.118 ± 0.027	0.120 ± 0.042	0.096 ± 0.027
User2	original	0.133 ± 0.031	0.109 ± 0.025	0.097 ± 0.016	0.123 ± 0.030	0.159 ± 0.021	0.099 ± 0.023	0.144 ± 0.024
	corrected	0.128 ± 0.097	0.085 ± 0.032	0.064 ± 0.014	0.054 ± 0.020	0.134 ± 0.021	0.145 ± 0.045	0.133 ± 0.037
User3	original	0.177 ± 0.038	0.183 ± 0.030	0.179 ± 0.021	0.214 ± 0.021	0.224 ± 0.032	0.262 ± 0.059	0.280 ± 0.085
	corrected	0.109 ± 0.036	0.130 ± 0.021	0.099 ± 0.018	0.092 ± 0.026	0.182 ± 0.054	0.167 ± 0.049	0.190 ± 0.053
User4	original	0.154 ± 0.041	0.173 ± 0.056	0.297 ± 0.024	0.335 ± 0.056	0.195 ± 0.033	0.225 ± 0.053	0.160 ± 0.061
	corrected	0.175 ± 0.031	0.190 ± 0.047	0.249 ± 0.030	0.282 ± 0.052	0.190 ± 0.039	0.237 ± 0.046	0.213 ± 0.073
User5	original	0.236 ± 0.049	0.192 ± 0.019	0.244 ± 0.029	0.200 ± 0.037	0.191 ± 0.033	0.240 ± 0.046	0.167 ± 0.065
	corrected	0.166 ± 0.033	0.198 ± 0.037	0.171 ± 0.018	0.156 ± 0.025	0.170 ± 0.040	0.242 ± 0.057	0.279 ± 0.040

**Table 3:** Velocity-DTW comparison for user 1 under Exp-B.

Joint	Original	Corrected	Improvement (%)
left_wrist	0.324	0.163	49.83
right_wrist	0.282	0.163	42.14
left_hip	0.041	0.022	46.93
right_hip	0.041	0.017	57.25
left_shoulder	0.174	0.038	78.03
right_shoulder	0.090	0.086	3.91
left_knee	0.105	0.059	43.63
right_knee	0.199	0.079	60.04
right_ear	0.130	0.047	63.76
Average	0.154	0.075	51.27

**Table 4:** Phase-wise Velocity-DTW statistics across users (Exp-B).

EXP-B		Address ~ Take_Away	Take_Away ~ Half	Half ~ Top	Top ~ Down_Half	Down_Half ~ Impact	Impact ~ Follow Through	Follow Through ~ Finish
User1	original	0.105 ± 0.047	0.170 ± 0.022	0.173 ± 0.020	0.141 ± 0.024	0.163 ± 0.024	0.220 ± 0.041	0.195 ± 0.024
	corrected	0.057 ± 0.026	0.072 ± 0.017	0.065 ± 0.019	0.089 ± 0.023	0.128 ± 0.032	0.130 ± 0.034	0.103 ± 0.015
User2	original	0.161 ± 0.038	0.139 ± 0.031	0.126 ± 0.020	0.163 ± 0.035	0.193 ± 0.029	0.126 ± 0.035	0.174 ± 0.024
	corrected	0.085 ± 0.045	0.096 ± 0.036	0.085 ± 0.023	0.076 ± 0.025	0.098 ± 0.019	0.118 ± 0.040	0.194 ± 0.029
User3	original	0.225 ± 0.048	0.232 ± 0.035	0.224 ± 0.023	0.268 ± 0.027	0.270 ± 0.038	0.295 ± 0.062	0.325 ± 0.082
	corrected	0.113 ± 0.043	0.124 ± 0.020	0.087 ± 0.019	0.119 ± 0.029	0.141 ± 0.023	0.189 ± 0.042	0.236 ± 0.057
User4	original	0.204 ± 0.054	0.217 ± 0.072	0.362 ± 0.034	0.452 ± 0.080	0.252 ± 0.041	0.261 ± 0.054	0.207 ± 0.075
	corrected	0.146 ± 0.049	0.138 ± 0.051	0.243 ± 0.035	0.313 ± 0.066	0.143 ± 0.031	0.137 ± 0.030	0.157 ± 0.040
User5	original	0.312 ± 0.063	0.246 ± 0.023	0.315 ± 0.037	0.256 ± 0.045	0.238 ± 0.031	0.283 ± 0.044	0.210 ± 0.076
	corrected	0.186 ± 0.098	0.166 ± 0.031	0.148 ± 0.031	0.140 ± 0.036	0.129 ± 0.037	0.217 ± 0.036	0.205 ± 0.037

**Table 5:** Comparison with non-learning baselines under consistent evaluation metrics.

Method	L2 Improvement (%)	Velocity-DTW Improvement (%)
Static L2 Minimization	28.7	4.9
DTW Alignment	9.8	42.1
Exp-A (RL *w*/*o* rhythm)	35.9	11.8
Exp-B (RL + rhythm)	47.1	51.3

**Table 6:** Ablation study of reward components under different configurations.

Model	Reward Terms	L2 Error	L2 Improv. %	V-DTW	VDTW Improv. %	Hip Drift
Exp-A	L2 + ΔL2 + Hip	0.078	35.91	—	—	0.035
Exp-B	L2 + ΔL2 + Hip + VDTW	0.063	47.06	0.075	51.27	0.032
Abl-1	Full − VDTW	0.107	10.15	0.204	11.87	0.04
Abl-2	Full − Hip	0.114	4.99	0.219	20.06	0.21
Abl-3	Full − ΔL2	0.126	5.73	0.228	25.23	0.133

**Table 7:** Reward-weight sensitivity analysis under different grid-search configurations.

Setting ID	α	β	λ	γ	Val-L2 Improv. %	Val-VDTW Improv. %	Val-Hip Drift	Notes
**S0**	**1.0**	**0.5**	**0.3**	**0.7**	**47.06**	**51.27**	**0.032**	**selected (best stability)**
S1	1.0	0.5	0.3	0.0	41.82	44.92	0.041	no rhythm term
S2	1.0	0.5	0.0	0.7	38.41	42.13	0.039	no hip term
S3	1.0	0.0	0.3	0.7	39.94	43.32	0.037	no improvement term
S4	1.5	0.5	0.3	0.7	45.25	48.68	0.035	stronger pose
S5	1.0	1.0	0.3	0.7	44.05	46.89	0.038	stronger smoothness
S6	1.0	0.5	0.6	0.7	43.33	45.55	0.036	stronger stability
S7	1.0	0.5	0.3	1.4	42.67	47.17	0.034	stronger rhythm

## Data Availability

The original contributions presented in this study are included in the article. Further inquiries can be directed to the corresponding author.

## Abbreviations

The following abbreviations are used in this manuscript:

RL	Reinforcement Learning
PPO	Proximal Policy Optimization
MDP	Markov Decision Process
DTW	Dynamic Time Warping
VDTW	Velocity-Based Dynamic Time Warping
GAE	Generalized Advantage Estimation
L2 Error	Euclidean Distance Error
Exp-A	Experimental Setting A (Static Pose Correction)
Exp-B	Experimental Setting B (Rhythm-Aware Correction)
2D	Two-Dimensional
KISTI	Korea Institute of Science and Technology Information
